# Reflections on mental healthcare for an asylum seeker population caught in limbo on the Greek island of Samos

**DOI:** 10.1192/bji.2022.3

**Published:** 2023-02

**Authors:** Lindsay Solera-Deuchar

**Affiliations:** MBBS, Psychiatrist, Medécins Sans Frontières, Vathy, Greece. Email: lindsay.soleradeuchar@gmail.com

**Keywords:** Refugee mental health, international work in psychiatry, transcultural psychiatry, post-traumatic stress disorder, depressive disorders

## Abstract

A psychiatry trainee reflects on a period of work on the Greek island of Samos with the international medical non-governmental organisation Medécins Sans Frontières/Doctors Without Borders, providing mental health and psychosocial support to asylum seekers. The clinic provided services to asylum seekers who were living in a crowded refugee camp, many of whom were experiencing symptoms of severe mental illness. The author reflects on the nature and severity of these presentations, and questions the role of psychiatry in treating mental illness that is clearly exacerbated by circumstances resulting from European asylum policies.

Like many medical students, I had dreamed of working for Médecins Sans Frontières/Doctors Without Borders (MSF) since I began my medical degree. When I discovered psychiatry, I thought that I would have to reconsider this dream, as I had never heard of psychiatrists working with MSF. But that was before I learned that mental health and psychosocial support is an integral part of MSF's work. Fast forward 12 years to November 2020, and with my core psychiatry training completed, I was on my way to the Greek island of Samos to work with MSF for the first time.

Samos is one of the five Greek islands with a reception and identification centre (RIC), created to host and ‘protect’ asylum seekers arriving from Turkey while their asylum application is being examined. Since the EU–Turkey deal in March 2016, Samos and the other islands have become bottlenecks in the journeys of asylum seekers to Europe, who are forced to spend indefinite periods on the islands waiting for their asylum claim to be processed.

The camp, just outside Vathy town, was originally built for around 600 people, but has hosted up to 10 times as many. During my time on Samos, there were an estimated 3500 people living in the camp. The original RIC is a small area in the centre of the camp, surrounded by an expansion of makeshift shelters, constructed by the asylum seekers themselves. Conditions are extremely crowded, with mountains of rubbish, rats and a constant need to repair shelters damaged by adverse weather. Before MSF started working there, water and sanitation facilities were woefully inadequate for the number of inhabitants. People from various countries in West, Central and East Africa and the Middle East all live in very close quarters, often without a language in common. My arrival was just 1 week after an earthquake, whose epicentre had been very close to Samos, as well as a series of fires in the camp, which had destroyed many people's tents, documents and other personal possessions.

## Mental healthcare and psychosocial support for the camp residents

On Samos, MSF provides mental health and sexual and reproductive health services at two day care centres outside the camp. I joined a team of four psychologists, several cultural mediators, a social worker and a mental health activity manager (who was also a psychologist). The aim of the project at that time was to provide quality mental healthcare and psychosocial support for asylum seekers, targeting those with more complex mental health needs. This was delivered through one-to-one and group sessions, with medication only for those with more severe symptoms. There were other organisations on the island providing mental health and psychosocial support, but overall psychologists and psychiatrists were in very short supply. Aside from two psychiatrists working at the public hospital, and a small handful of private practitioners, I was the only psychiatrist on the island.

In theory, asylum seekers have the right to access the public mental health services, but the mental health department was overwhelmed and getting an appointment was extremely difficult. There were no in-patient psychiatric facilities on the island, and arranging an admission on the mainland was next to impossible and only considered in extreme circumstances.

## Common presentations in the clinic

My role consisted of seeing patients referred by the psychologists for new assessments and follow-up appointments. I was immediately struck by the severity of mental illness in these individuals, and had to quickly readjust my expectations of risk management, owing to the limited options for follow-up outside the clinic. Seeing the ongoing uncertainty, the abysmal living conditions and the lack of sense of safety that people had to live with, I also had to change my expectations of what we could achieve: aiming for complete recovery was totally unrealistic. As the medical team leader told me, we were aiming to ‘keep people afloat’ until they reach a place where they can begin to recover.

Many of the patients I cared for had suicidal thoughts, and most had either depressive symptoms, post-traumatic stress disorder (PTSD) symptoms or, very often, a combination of the two. Something I had rarely encountered before in my psychiatry training became very routine: secondary psychotic symptoms in PTSD. I began to question the concept of psychosis I had gained from my training. In my mind, psychosis was usually associated with the thought disorder, disorganisation and impairment of functioning that is often seen in schizophrenia. However, these individuals were describing very clear psychotic symptoms, with auditory hallucinations and persecutory delusions, yet were functioning relatively well and had more insight than I would have expected. With the help of some reading around the topic, I began to understand psychosis better in the context of dissociation, including the likely overlap between the two phenomena.

People's presentations and expressions of distress were, of course, influenced by their culture, personal experiences and current social circumstances. Although each person's experience is unique and not generalisable, I did notice some patterns. There were several single men from Central and West African countries experiencing quite severe PTSD symptoms, with secondary psychotic features. Many women from the Middle East, who had travelled to Samos with their families, expressed what I interpreted as a depressive illness through multiple physical symptoms and irritability with their children and husbands. I met multiple young men from Central and West African countries who had experienced what seemed to be a functional developmental regression in response to trauma. Their daily lives were now dependent on the support of family members and fellow camp residents.

Expectations of treatment also varied. Several patients from the Middle East had previously been prescribed medication in their own countries, such as antipsychotics for sleep, and hoped for the same from our service. Many patients from West and Central Africa had never had any contact with mental health services before and found the idea of having a say in their treatment very unusual. Our cultural mediators, many of whom were themselves asylum seekers, were indispensable.

## Reflections on the role of psychiatry in this context

I reflected on why these patients were presenting with such severe mental illness. Many had experienced traumatic events in their countries of origin or *en route* to Greece, including war, torture, trafficking and sexual violence. But my feeling was that the situation that they found themselves in on Samos played a big part in exacerbating their symptoms. Many had fled their countries with the hope that they could find a better life in Europe and put these traumatic experiences behind them. However, after being brought to Samos, they found themselves living in dehumanising conditions, in a state of limbo, not knowing if their asylum claim would be accepted or if they would be sent back to face the horrors they had fled. Moreover, to be able to recover from past traumatic experiences, a sense of safety is paramount, and in the crowded conditions of the camp, with no lockable doors and periodic fires, this was impossible.

There were times when I doubted if we were really helping at all. I often felt very helpless, such as when I saw patients who were in acute distress following an asylum rejection, expressing that there was no point in going on living. When I explored my patients’ sleeping difficulties and learned that the noise in the camp, along with fear of fires in the night and rats crawling around their tents, were the main issues, I was at a loss for how to help.

However, as the time went on and I saw small improvements in my patients, I began to understand why MSF was there. Although the medication was helping to ease their symptoms a little, the simple fact that they had somewhere to go on a regular basis where they felt safe and listened to probably made the greatest difference. When my time in Samos came to an end, several patients expressed how much our support had mattered to them.

My assignment in Samos was the most emotionally challenging work I had done in my psychiatric career so far. Being with people experiencing this kind of distress daily was hard. I had never before needed to make such an effort to take care of my own mental well-being. Living close to the camp made it difficult to switch off from work. Whenever there was heavy rain I would picture my patients trying to stop the rain coming into their tents and wish that there was more that we could do to help. But in many ways it felt very important to bear witness to the dire experiences that so many asylum seekers sadly have to endure. I hugely admire their resilience and ability to retain some hope in the face of such prolonged uncertainty. My hope is that we as the Western world can move away from asylum policies that have such damaging effects on mental health and well-being, because to me it's clear that psychiatry isn't the solution.

## Data Availability

Data availability is not applicable to this article as no new data were created or analysed in this study.

